# Two Cases of Accidental Wound of the Femoral Artery

**Published:** 1893-01

**Authors:** E. Dickson

**Affiliations:** Coal Creek, Tenn.


### TWO CASES OF ACCIDENTAL WOUND OF THE FEMORAL ARTERY.*

By E. DICKSON, M. D.,

COAL CREEK, TENN.

I will not consume time by giving a long and complete history of these cases, but will present them as briefly as possible. They are only interesting in showing that wounds of large arteries will sometimes heal by natural processes as in Case No. 1; and Case No. 2 shows how an incised wound of the femoral artery may heal perfectly with the use of only one ligature.

Case No. 1 represents an unusual result of a gunshot wound of a large artery; it is briefly as follows: A boy about ten years of age received a wound from a 22 calibre rifle fired at close range. The ball entered the groin of the left side about one-half inch below Poupart's ligament and directly over the femoral artery, traversed the pelvis and made its exit through the buttock at a point near the anus.

There was a very free hemorrhage but it was promptly controlled by pressure made with the hands. When the patient was seen by me in consultation with my colleague, Dr. McFerrin, hemorrhage had ceased but there was evidence of considerable shock. A light compress was applied over entrance wound and the patient watched closely for several hours, when, all being well, he was left with his attendants. Although it was found to be impossible to keep the boy quiet there was no more hemorrhage.

A small traumatic aneurism developed promptly with distinct thrill and bruit. Contrary to my expectations the aneurism soon began to grow less and finally disappeared altogether. There was no suppuration about the wound and the patient made a rapid recovery, though it was sometime before I could be convinced

*Read before the Tri-State Medical Society, Chattanooga, Tenn., Oct., 1892.

that the patient would not develop a diffuse aneurism that would require operative treatment; but as nearly two years have now elapsed since the wound was inflicted I have long since ceased to regard this boy as a promising candidate for the operating table.

In this case I fully expected to have to ligate, but realizing the great danger of ligating at this point under such circumstances (owing to the proximity of large branches) I determined to wait until the operation was imperative. The result justified the method pursued, though such a result was not to be expected when we take into consideration the history of such cases; but it impresses on us the lesson, not to be too hasty with our operative measures.

I have not seen mentioned another instance where recovery without operation followed such a severe wound of so large an artery.

Another case that occurred in the practice of a brother physician represents a not unusual result of a gunshot wound of an artery, and is as follows:

A young man received a pistol-shot wound in the lower third of the thigh; hemorrhage very free, controlled by bystanders tightly cording the limb. Hemorrhage did not return and the patient was soon going about apparently perfectly recovered. A few months later he presented himself with a large pulsating tumor at point of late injury. The femoral artery was tied in Scarpa's space and patient made a good recovery.

CASE No. 2.—A boy about eight years of age received a puncture of the femoral artery by the sharp point of a knife, the cut being made in a longitudinal direction.

Hemorrhage was profuse, but was promptly checked by pressure of finger in the wound. I then applied an Esmarch's cord about the thigh and placed a ligature around the artery well above the wound. This was done with considerable difficulty as it was night and light was derived from a lard oil lamp; moreover I had but one assistant, and he was fully occupied in administering chloroform.

I could not see the wound of the artery nor anything very distinctly, but principally by the sense of touch passed the ligature around the artery. Finding that the single ligature controlled the hemorrhage perfectly, I determined under the circumstances to omit the second ligature, for the time being, at least. An abscess complicated matters, and the wound had to be left open for drainage. For several days I feared a return of hemorrhage from the distal side of the wound, but the patient made a good recovery.

In such cases it is undoubtedly safer to use the double ligature, but it is evident that even an incised wound of the femoral artery, and complicated by unfavorable conditions, can heal perfectly with only one ligature. From the result in these two cases we may infer that in wounds of arteries we may, under some circumstances, be justified in resorting to temporizing measures not in accordance with well established rules of surgery, and we see how much nature can do to control hemorrhage and hea these wounds.

